# Blindness to Curvature and Blindness to Illusory Curvature

**DOI:** 10.1177/2041669518776986

**Published:** 2018-05-24

**Authors:** Marco Bertamini, Akiyoshi Kitaoka

**Affiliations:** Department of Psychological Sciences, University of Liverpool, UK; Department of Psychology, Ritsumeikan University, Kyoto, Japan

**Keywords:** curvature, visual illusion, contrast polarity, shape, contour curvature

## Abstract

We compare two versions of two known phenomena, the *Curvature blindness* and the *Kite mesh* illusions, to highlight how similar manipulations lead to blindness to curvature and blindness to illusory curvature, respectively. The critical factor is a change in luminance polarity; this factor interferes with the computation of curvature along the contour, for both real and illusory curvature.

In a recent paper, Takahashi (2017) reported a striking novel illusion. The curvature of a sinusoidal line becomes hard to see, and observers perceive a zig-zag (straight segments). In Figure 1, we show a sinusoidal line, as lighter than the grey background, darker than the background, or a mixture of light or dark segments. Figure 2 shows a sinusoidal line with a different type of segmentation. Here, the change from dark to light happens at the peak of the curve (curvature extrema) instead of at the inflection point. Note that in Figure 2, perceived curvature is strongly reduced. Takahashi called this the *Curvature blindness* illusion. He concluded that the mechanism for perception of smooth curvature and that for perception of corners compete with each other and the corner percept might be dominant.

**Figure 1.**
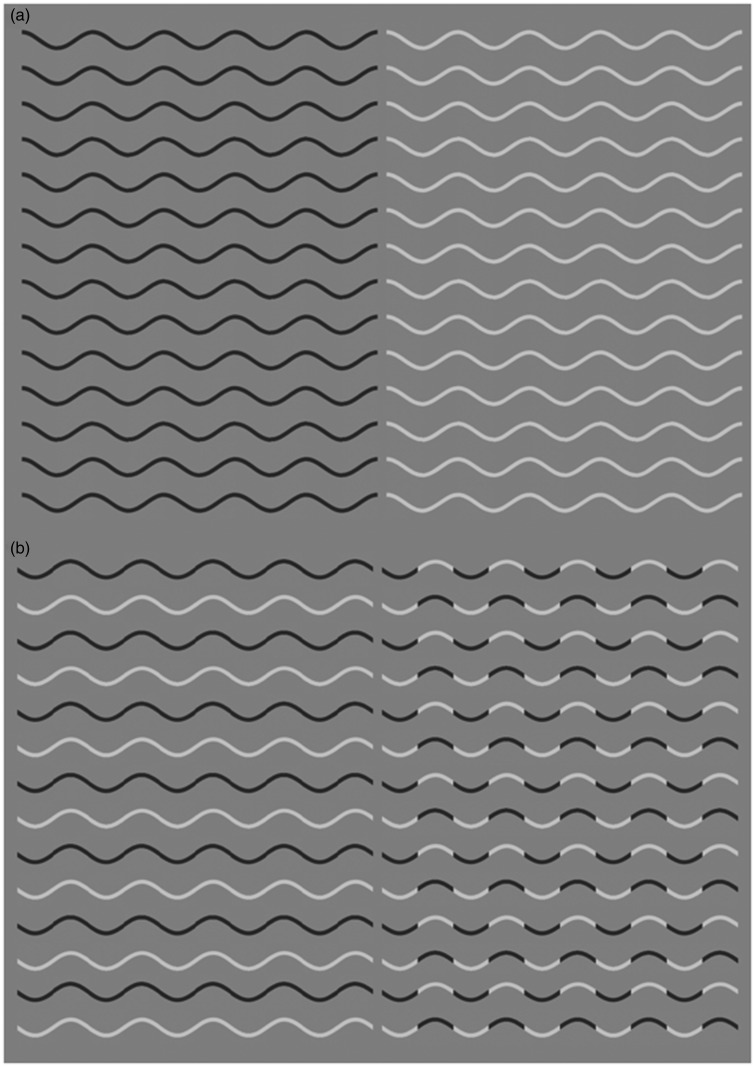
(a) Here, we show sinusoidal lines either lighter or darker than the background. (b) These are a combination of the light and dark lines, including a case in which the line changes colour at the point of inflection. The *Curvature blindness* illusion is shown in Figure 2, and these are images for comparison, so if you do not see any illusion here, that is how it should be.

**Figure 2.**
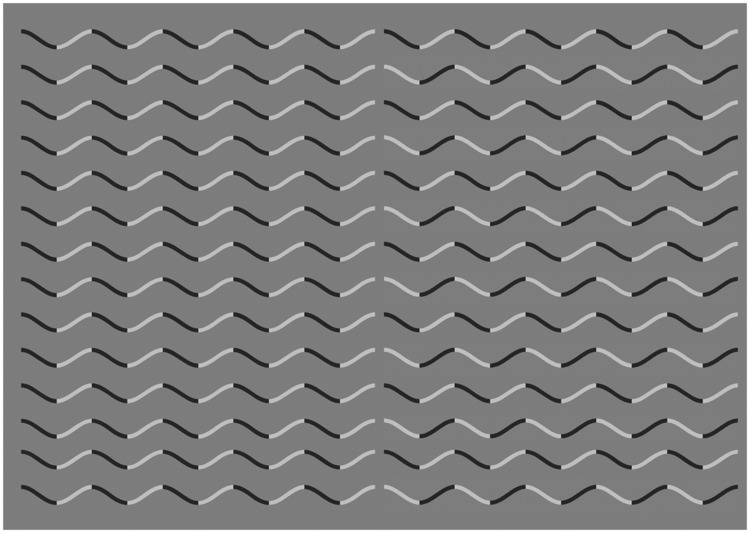
*Curvature blindness* illusion. Here, we show sinusoidal lines either lighter or darker than the background. Because the change is at the turning points (curvature extrema), the perceived curvature is greatly reduced (Takahashi, 2017).

Based on the study by Takahashi (2017), it is interesting to note that the segmentation has to be a change in contrast polarity, which means that the change along the contour has to be in opposite direction compared to the background. Simply changing the brightness would not be sufficient for the effect.

## Processing Curvature

One way to describe the *Curvature blindness* illusion is in terms of average contour along the segments. Given that the visual system can only detect orientation at discrete locations, curvature is derived as an integrated property over some length of a contour. For instance, Blakemore and Over (1974) suggested that curvature is extracted ‘by reference to the orientations of local straight-line approximations within the arc, rather than through immediate global analysis of the extent and direction of curvature per se’ (p. 3). Formal models of contour integration and of how curvature is detected incorporate this idea and rely on local edge orientation (Wilson, 1985; Wilson & Richards, 1989). Some studies have shown that gaps and line intersections along a contour impair perception of curvature (Watt, 1984, 1985). Using a contour completion task, Field, Hayes, and Hess (2000) compared contours created by Gabor patches that did or did not alternate in luminance polarity (a difference in phase) and found evidence for sensitivity to polarity.

Many studies, starting with Blakemore and Over (1974), have used shape adaptation to study the perception of curvature. In a series of experiment, Gheorghiu and Kingdom (2006, 2007) have found that aftereffects of curvature are selective to luminance polarity. More recently, Bell, Gheorghiu, Hess, and Kingdom (2011) have studied adaptation of global shapes and of components patches. They found that changes in luminance polarity of the patches disrupts adaptation and affects sensitivity. They concluded that the mechanisms that process contours are polarity selective.

In the study of orientation, using Gabor patches, it has been observed that neighbouring Gabors greatly affect the sensitivity to the orientation of a target patch (Solomon, 2010). This supports the view that some computations are automatic, and therefore observers have only access to average values, in this case orientation.

Returning to the examples of *Curvature blindness*, when the segmentation happens following the pattern of Figure 2, each resulting segment has an inflexion point in the middle and, on average, its curvature is zero. It is shown in Figure 2 that each segment has the shape of an S. Therefore, it is possible that certain properties of the stimulus segment a contour in a way that integration can only take place within individual segments. To support the idea of a competition between smooth curvature and corner perception, Takahashi (2017) also makes a link between the *Curvature blindness* illusion and another effect, reported by Ito (2012). Ito found that adaptation to a circle produces an afterimage with the shape of a hexagon. In addition, even the prolonged observation of a circle is enough to make the stimulus deform into a polygon. This is a striking effect that deserves more research; it is mentioned by Takahashi because it illustrates a possible competition in the brain between representations of smooth curvature and corners.

## Two Illusions, One Mechanism

**Figure 3.**
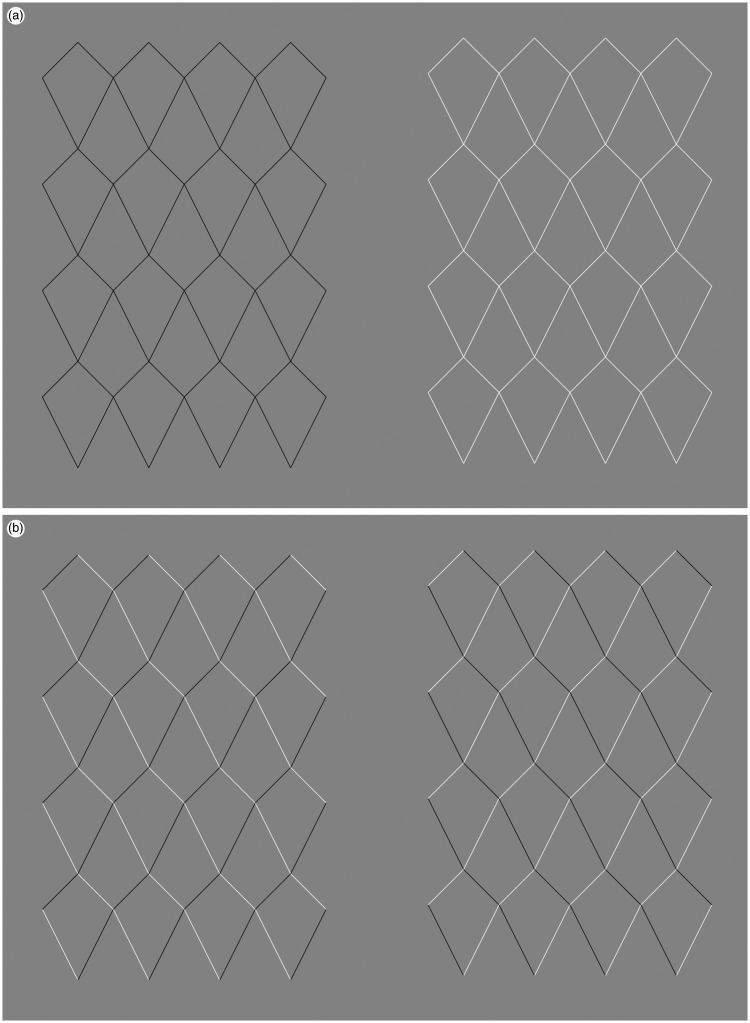
The *Kite mesh* illusion. (a) Two versions of the illusion in which the diagonal lines appear to have some curvature, despite the fact that only straight lines are used to create the pattern (for the original, see www.psy.ritsumei.ac.jp/∼akitaoka/friends10e.html). (b) The illusion works also with a mix of white and black lines, as long as there is no colour change in the direction of continuation of the contour.

We are going to discuss a parallel between the *Curvature blindness* illusion and another illusion. Yoshiko Ishibashi published this illusion on Akiyoshi Kitaoka’s website (www.psy.ritsumei.ac.jp/∼akitaoka/friends10e.html). The caption says ‘Kite mesh illusion. Each side of a quadrangle is linear, but it appears to be distorted. Copyright Yoshiko Ishibashi 2014 (June 5)’. Four versions of the illusion are shown in Figure 3. For light, dark or a mixture of light and dark lines, there is an illusory bending of the oblique lines (top left to bottom right and vice versa). When the lines going toward the right are of a different colour compared to the lines going to the left (Figure 3, panel (b)), the bending illusion may even appear stronger.

These stimuli have lines that are thinner than the lines used in the *Curvature blindness* illusion (Figures 1 and 2) because that is necessary for the effect.

The *Kite mesh* illusion belongs to a family of illusions in which straight lines appear to bend. Indeed, the very first illusion that was published using the terminology of ‘optical-geometrical illusion’, belongs to this family. The paper was published in 1855 by Oppel and the illusion consisted of three straight lines, the line intersected by the other two appears to bend slightly. A bending of straight lines is also present in the Hering, Orbison and Bourdon illusions (see Vicario, 2011, or Bertamini, 2017).

**Figure 4.**
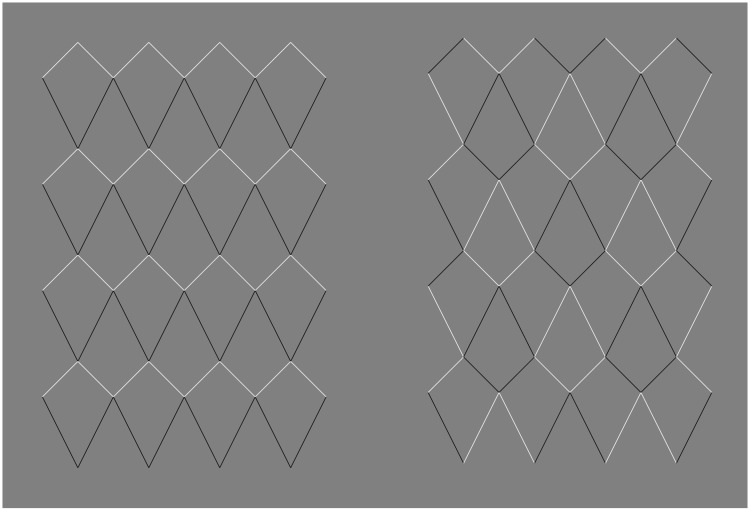
No curvature is visible when the lines change brightness along the direction where curvature is perceived. This is true for two different configurations (left and right), which create different effects in terms of 3D, grouping, and different perceived illumination.

Figure 4 shows that the percept of curvature is lost when there is a change of luminance polarity along the contour. Note how this is true in both versions of Figure 4, suggesting that the critical factor is the change of luminance polarity at the intersections along the line, and not the specific grouping formed by the different colour lines. In the left case, for instance, there is a possible percept of light coming from above, but this is not possible in the right version.

**Figure 5.**
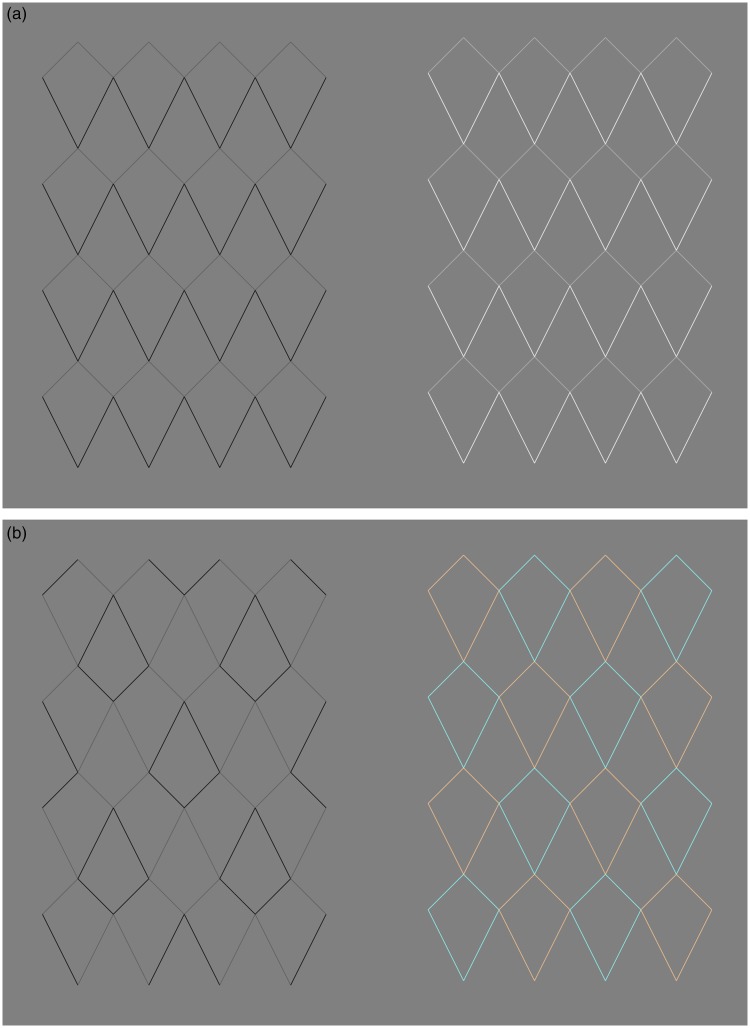
These are not parametric manipulations, but the four examples support the view that polarity is important, as other changes (segmentation by change of colour/luminance) do not eliminate the illusion of curvature.

**Figure 6.**
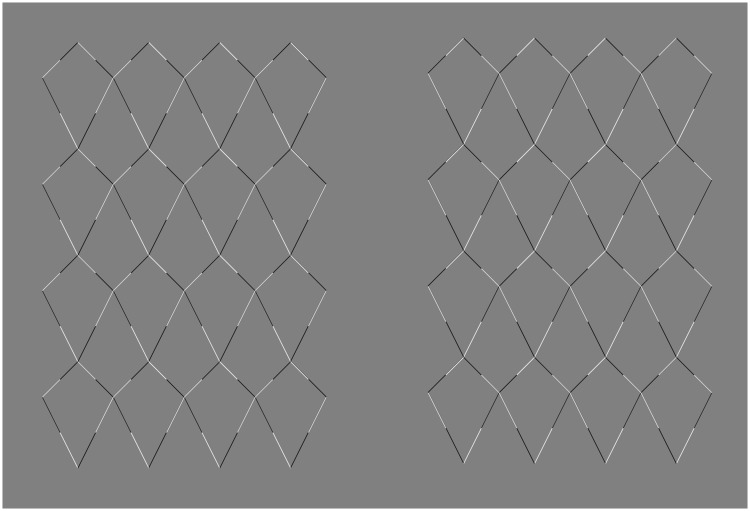
Here, polarity changes midway along the segment. This image was designed to be similar to the manipulation in Figure 1 (last panel). The perceived curvature in this case is strong.

**Figure 7.**
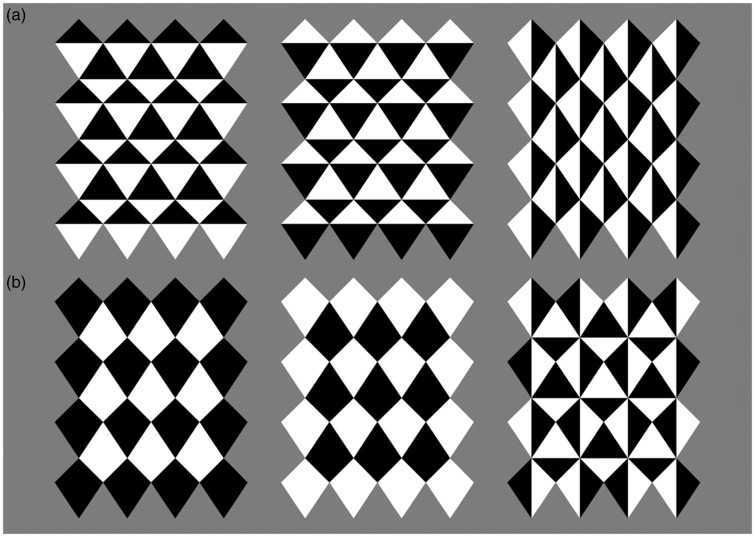
The *Kite mesh* illusion, the tile version. (a) Some bending is visible in these three configurations, despite the changes of luminance and the tessellation. The critical factor is that along the oblique line the contour has always white on the right and black on the left, or vice versa. (b) Here, there is no perceived curvature because along the contour a white or black segment is followed by a black or white segment, and vice versa.

Figures 5 to 7 explore the perception of bending in the *Kite mesh* illusion with changes of luminance or colour and by changes of the colour of the entire tiles. Some curvature is perceived in all the cases of Figure 5, and in the cases of Figure 6, the effect is strong. The changes in Figure 5 are changes at the end of the segments, but they do not involve a change of luminance polarity. This suggests that polarity is important and other changes do not entirely eliminate the illusory curvature. Figure 6 is similar to a control condition of the *Curvature blindness* effect that we have already seen in the last panel of Figure 1. As the change of luminance happens midway along the segments, this does not affect the real or illusory curvature. It seems that in the case of Figure 6 this change may also contribute to the illusion.

Figure 7 provides a different type of manipulation. We called this the tile version of the *Kite mesh* illusion. The tiling in itself does not eliminate the illusion. This is interesting because the tiling does have an effect on the perceived three-dimensional structure of the surfaces. All the tiles appear flat. With respect to the curvature perceived in the top three examples, the critical factor is that along the oblique line the contour has always white on the right and black on the left, or vice versa. However, in the bottom three examples, there is less or no perceived curvature because along the contour a white or black segment is followed by a black or white segment, and vice versa.

## Legibility

**Figure 8.**
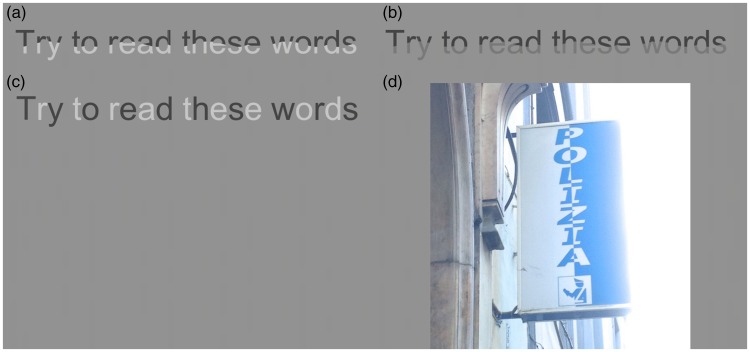
Changes of polarity along the contours of letters may be detrimental for legibility. The change of polarity in panel (a) can be compared to changes of luminance in panel (b). Note that panel (b) has lower contrast between letters and background (for the lower part of the letters). In panel (c), we have changes of polarity between letters. In panel (d), there is a photo of a sign outside an Italian police station. We find this sign aesthetically pleasing but not easy to read (photo by MB).

Figure 8 provides a final example. If changes of polarity affect contour integration, they may also affect the legibility of a sentence. In the first panel of Figure 8, polarity changes abruptly along the contour of the letters. This can be compared to a change of luminance (panel (b)) or a change of polarity between letters (panel (c)). For the change of luminance, we have kept the contrast the same (top part) or lower (bottom part) than in the other cases so that presumably the advantage of panel (b) over panel (a) cannot be attributed to contrast. Although we are not aware of a direct study of polarity change along the contour of the letters, work on the effect of noise, transparency, and of matrix-sampled text are relevant (Legge, Pelli, Rubin, & Schleske, 1985; Scharff & Ahumada, 2002). There are also studies comparing dark letters on light background and vice versa, showing an advantage for the former case (Scharff & Ahumada, 2008), and Chung and Mansfield (2009) have studied the case of a luminance polarity change between letters (similar to panel (c) of Figure 8). They found that, despite the fact that this change of polarity between elements reduces crowding, it has no effect on legibility. Note that this change is not along the contour of the letter and is therefore not expected to affect the processing of curvature.

## Conclusions

In the case of the *Curvature blindness* illusion, the misperception of curvature emerges when there is a change of luminance polarity at the turning point. Integration of contour curvature information is constrained by polarity. The curvature for each of the segments, when they are processed separately, is misperceived in the direction of the average curvature, with a lack of integration across the boundary.

We agree with Takahashi (2017) that the *Curvature blindness* illusion is informative about how curvature is perceived, and there is a literature on perception of shape when contour polarity is manipulated, in particular in the case of adaptation and aftereffects (e.g. Bell et al., 2011). There may also be a link with perception of shape more generally. Contour curvature, extrema and convexity are long been understood to be important properties in shape perception and in relation to solid shape (Bertamini & Wagemans, 2013; Koenderink, 1984).

The *Kite mesh* illusion offers us a complementary situation. Here, we start from an illusion: the curvature along the contour is misperceived when there are intersections (but no segmentation by polarity). This distortion may result from properties of the mechanism involved in integrating curvature along a contour. The phenomenon of straight lines perceived as curved is consistent with other optical-geometrical illusions, such as the Oppel illusion.

We have observed that in the *Kite mesh* illusion there is blindness to the illusory curvature when the contour is segmented. The change of luminance polarity with respect to the background has the effect of interfering with the process of curvature perception. This effect is the same as in the *Curvature blindness* effect, but in the case of the *Kite mesh* illusion, since curvature is illusory, the change in polarity makes the straight lines appear straight. Combining the observations about the *Curvature blindness* and the *Kite mesh* illusions strengthen the argument that luminance polarity has a key role on the process of curvature computation along a contour.
